# Room Temperature Stable PspA-Based Nanovaccine Induces Protective Immunity

**DOI:** 10.3389/fimmu.2018.00325

**Published:** 2018-03-02

**Authors:** Danielle A. Wagner-Muñiz, Shannon L. Haughney, Sean M. Kelly, Michael J. Wannemuehler, Balaji Narasimhan

**Affiliations:** ^1^Department of Veterinary Microbiology and Preventive Medicine, Iowa State University, Ames, IA, United States; ^2^Department of Chemical and Biological Engineering, Iowa State University, Ames, IA, United States; ^3^Nanovaccine Institute, Iowa State University, Ames, IA, United States

**Keywords:** pneumococcal infections, pneumococcal surface protein A, polyanhydride, nanovaccine, room temperature stability

## Abstract

*Streptococcus pneumoniae* is a major causative agent of pneumonia, a debilitating disease particularly in young and elderly populations, and is the leading worldwide cause of death in children under the age of five. While there are existing vaccines against *S. pneumoniae*, none are protective across all serotypes. Pneumococcal surface protein A (PspA), a key virulence factor of *S. pneumoniae*, is an antigen that may be incorporated into future vaccines to address the immunological challenges presented by the diversity of capsular antigens. PspA has been shown to be immunogenic and capable of initiating a humoral immune response that is reactive across approximately 94% of pneumococcal strains. Biodegradable polyanhydrides have been studied as a nanoparticle-based vaccine (i.e., nanovaccine) platform to stabilize labile proteins, to provide adjuvanticity, and enhance patient compliance by providing protective immunity in a single dose. In this study, we designed a room temperature stable PspA-based polyanhydride nanovaccine that eliminated the need for a free protein component (i.e., 100% encapsulated within the nanoparticles). Mice were immunized once with the lead nanovaccine and upon challenge, presented significantly higher survival rates than animals immunized with soluble protein alone, even with a 25-fold reduction in protein dose. This lead nanovaccine formulation performed similarly to protein adjuvanted with Alum, however, with much less tissue reactogenicity at the site of immunization. By eliminating the free PspA from the nanovaccine formulation, the lead nanovaccine was efficacious after being stored dry for 60 days at room temperature, breaking the need for maintaining the cold chain. Altogether, this study demonstrated that a single dose PspA-based nanovaccine against *S. pneumoniae* induced protective immunity and provided thermal stability when stored at room temperature for at least 60 days.

## Introduction

Community-acquired pneumonia is a debilitating disease and is globally responsible for 16% of deaths in children under the age of five annually (1). *Streptococcus pneumoniae*, a causative agent of bacterial pneumonia, is a Gram-positive bacterial pathogen of humans, which colonizes the upper respiratory tract and leads to the development of potentially life-threatening diseases, such as otitis media, sinusitis, and pneumonia, with pneumonia being the most serious of these conditions (2, 3). While otitis media and sinusitis are not as severe, they still account for seven million cases in the United States annually (2). *S. pneumoniae* is asymptomatically carried in approximately 25% of healthy individuals and is spread between individuals through respiratory droplets, with children serving as the primary transmission source to adults (4–7). Pneumococcal infections are most prevalent in infants, young children, and the elderly, accounting for over $3 billion spent annually in direct healthcare spending; in addition, 60–87% of all pneumococcal bacteremia cases are associated with pneumonia in adults (2). While control of *S. pneumoniae* by antibiotics has been beneficial, up to 50% of all strains are resistant to erythromycin, with 97% of those also being resistant to azithromycin, making intervention strategies such as vaccination a necessary component of health care management (8). To date, the single most effective advance in the field of pneumonia prevention has been through immunization, though existing vaccines are still not completely effective (9, 10).

Current preventive strategies involve the use of pneumococcal polysaccharide vaccines (PPVs) and pneumococcal conjugate vaccines (PCVs). PPVs (T-independent antigens) comprise capsular polysaccharides, which are poorly immunogenic in very young and elderly individuals, while PCVs (T-dependent antigens) are more effective in these at-risk populations because of the adjoined protein component. While the introduction of PPV23, PCV7, and PCV13 have had significant contributions in reducing the overall global burden of pneumococcal pneumonia, there is still room for improvement (11). As it currently stands, infection with strains not covered by vaccine serotype infections account for approximately 50% of all deaths in individuals over 55 years of age (12). In addition, it has been observed that introduction of new vaccine strategies against *S. pneumoniae*, such as the introduction of PCV10 immunization program for children in the Netherlands or use of PCV7 in Spain, may be directly correlated to an increased emergence of disease caused by non-vaccine serotype strains cases (13–15). It was demonstrated that PCV7 immunization, which contains the 19F serotype antigen, actually caused an increase in pneumococcal infections by the closely related 19A serotype (16). Globally, this is of particular concern when considering the development of a universal vaccine, as it is now clear that the prevalence of certain serotypes differs greatly between age, geographic region, and ethnicity (3, 17, 18). PPV23 encompasses the most pneumococcal serotypes of the commercially available vaccines. However, it is not recommended for use in children under the age of 18 months because of their poor antibody response to T-independent polysaccharide antigens, explaining why PCV13 is recommended for this age group. While the use of PCV and PPV vaccine formulations have effectively reduced the incidence of pneumococcal pneumonia, there is a need for newer vaccines to protect against the emergence of non-vaccine strains of *S. pneumoniae* (19). Coupled with the phase variation in expression of capsular and surface protein antigens, broadly protective vaccines against colonization and invasive pneumococcal disease are likely to require the inclusion of more conserved antigens that will also facilitate the induction of antigen-specific CD4^+^ T cells (20, 21).

*S. pneumoniae* has several key virulence factors that play a critical role in colonization, transmission, and tissue damage, including pneumolysin, two neuraminidases, and pneumococcal surface protein A (PspA). PspA is a choline binding protein, one of the most abundant proteins located on the pneumococcal cell surface, and has been shown to be of particular importance in facilitating nasopharangyl colonization through inhibiting host complement responses (22). In the design of new vaccines against *S. pneumoniae*, it is important to consider a target immunogen that will be capable of inducing a broad, serotype-independent, protective immune response. PspA has been of recent interest as a potential vaccine candidate due to its location on the cell surface of all 95 strains of *S. pneumoniae* currently known and its critical role in pneumococcal pathogenesis (3, 23, 24). Anti-PspA antibodies to clades 1 and 2 of PspA have been shown to be cross-protective against *S. pneumoniae* strains encompassing all six clades of PspA, and provide protection when passively transferred to naïve mice as a therapeutic intervention following septic *S. pneumoniae* challenge (17, 25–27). The ability to provide protection across many serotypes of *S. pneumoniae* using only two clades of PspA allows for the design of a broadly cross-protective vaccine when compared to vaccines containing numerous capsular polysaccharides.

Polyanhydride nanoparticle-based vaccines (i.e., nanovaccines) represent next-generation vaccine platforms against pathogens such as *S. pneumoniae*. These nanovaccines are formulated using random copolymers based on 1,8-bis-(p-carboxyphenoxy)-3,6-dioxaoctane (CPTEG), 1,6-bis-(p-carboxyphenoxy)hexane (CPH), and sebacic acid (SA) and have been extensively studied as a nanovaccine platform against infectious pathogens, such as influenza virus, *Yersinia pestis*, and *Bacillus anthracis* (28–31).

Polyanhydrides provide vaccine delivery benefits and adjuvant properties that make them well suited as a vaccine delivery platform. These materials exhibit high biocompatibility with minimal injection site reactivity (32–34) (i.e., tenderness, swelling, and pain) in comparison to traditional adjuvants, such as Alum, which has been associated with immunization site tenderness and pain, and are currently FDA-approved for use to treat malignant gliomas in the brain (35, 36). In addition, polyanhydrides are hydrophobic and exhibit surface erosion characteristics, which helps stabilize labile proteins, protects protein denaturation from enzymatic cleavage and acidic degradation products, and allows for the prolonged release of antigen (28, 37–41). The sustained release of antigen allows for enhanced bioavailability of antigen to drive adaptive immune responses and allows for single dose administration and dose-sparing capabilities (30, 42–44). Previous work has shown that mice immunized with a single dose of nanovaccine encapsulating *Y. pestis* fusion protein F1-V were completely protected against *Y. pestis* lethal challenge after at least 280 days post-immunization (DPI) (31). Varying polyanhydride copolymer composition has shown to modulate internalization and persistence within APCs *in vitro*, as well as induction of both cellular and humoral immune responses *in vivo* indicating the ability to tailor polymer chemistry in order to rationally design nanovaccines that optimally inducing antigen-specific protective immunity (31, 42, 45–54).

Previous work from our laboratories has demonstrated that PspA encapsulated into nanoparticles maintained its stability, conformational structure, as well as biological activity upon release, which was measured using an apolactoferrin binding assay (37). In this same work, mice immunized with a single dose of the nanovaccine generated robust antibody titer and avidity to PspA, equivalent to that of antigen adjuvanted with MPLA. However, the protective efficacy of a PspA-based nanovaccine against lethal challenge has not been demonstrated. In this study, the ability of various polyanhydride nanovaccine chemistries as vaccine candidates was evaluated and it was demonstrated that a lead PspA-based nanovaccine protected animals against lethal challenge with a 25-fold reduction in overall PspA dose. Furthermore, this lead formulation was shown to be capable of storage at room temperature for at least 2 months without any loss of efficacy.

## Materials and Methods

### Materials

Chemicals used for monomer and polymer synthesis included sodium hydroxide, hydrobenzoic acid, dibromohexane, 1-methyl-2-pyrrolidinone, SA monomer, and triethylene glycol purchased from Sigma Aldrich (St. Louis, MO, USA). Acetic anhydride, ethyl ether, petroleum ether, chloroform, methylene chloride, hexane, acetone, sulfuric acid, potassium carbonate, dimethyl formamide, toluene, acetonitrile, N,N-dimethylacetamide, and acetic acid were purchased from Fisher Scientific (Fairlawn, NJ, USA); 4-p-fluorobenzonitrile was purchased from Apollo Scientific (Cheshire, UK). For ^1^H NMR analysis of the copolymers, deuterated chloroform and dimethyl sulfoxide were purchased from Cambridge Isotope Laboratories (Andover, MA, USA). The N-terminal region of a recombinant PspA (UAB055, PspA/Rx1 AA1 to 303, clade 2 PspA of the PspA family 1) was produced by Dr. David McPherson (University of Alabama at Birmingham) as described previously (55). Prior to immunization, endotoxin was removed from the protein using endotoxin removal beads (Miltenyi Biotec, Bergisch Gladbach, Germany) according to the manufacturer’s instructions followed by dialysis and lyophilization. The final endotoxin content of the protein was determined to be less than 1.9 EU/mg as determined by a limulus amebocyte lysate chromogenic endotoxin quantification kit (Thermo Fisher Scientific).

### Copolymer and Nanoparticle Synthesis

Monomers of CPH and CPTEG were synthesized as previously described (28, 49). A 50:50 molar composition copolymer of CPH and CPTEG and a 20:80 molar composition copolymer of CPH and SA was synthesized using melt condensation as described by Torres et al. (28). PspA protein, 1% (w/w), was encapsulated into polyanhydride nanoparticles using solid/oil/oil nanoprecipitation, as previously described (37). Excess buffer was removed from PspA protein solution using a 5 kDa MWCO dialysis microcentrifuge tube and the resulting protein solution was lyophilized overnight at −40°C under vacuum. The lyophilized protein and the respective copolymer was then dissolved in methylene chloride at a concentration of 20 mg/mL of solvent. The solution was sonicated using the VibraCell ultrasonic probe (Sonics & Materials, Inc., Newtown, CT, USA) to ensure complete dissolution and homogenization of the protein and the copolymer. The resulting solution was then added to pentane at a 1–200 (v/v) ratio of methylene chloride to pentane at 20°C for the 20:80 CPH:SA nanoparticle formulation or at a 1–250 (v/v) ratio of methylene chloride to pentane at −40°C and for the 50:50 CPTEG:CPH nanoparticle formulation. Nanoparticles were collected using vacuum filtration. Surface charge of representative 20:80 CPH:SA and 50:50 CPTEG:CPH nanoparticle formulations were measured using quasi-elastic light scattering (Zetasizer Nano, Malvern Instruments Ltd., Worcester, UK).

### Animals

CBA/CaHN-Btkxid/J (CBA/N) mice were purchased from Jackson Laboratory (Bar Harbor, ME, USA) and used for the immunization studies. These mice have a mutation in their Bruton’s tyrosine kinase gene. Due to this mutation, these mice are unable to initiate a proper antibody response to Type II thymic-independent antigens (e.g., capsular polysaccharides), and are, thus, able to better mimic natural antibody responses in at-risk elderly or young populations. Mice were housed under specific pathogen-free conditions where all bedding, caging, water, and feed were sterilized prior to use. All studies were conducted with the approval of the Iowa State University Institutional Animal Care and Use Committee.

### Immunization Protocols

Groups of 8–10 CBA/N mice were immunized subcutaneously at the nape of the neck with 0–20 µg PspA, 0–5 µg PspA encapsulated into 50–500 µg polyanhydride nanoparticles, and/or 50 µL of Imject Alum Adjuvant (ThermoFisher Scientific) and delivered in a total volume of 100 µL. Immunization groups for the individual studies (see Tables 1 and 2) comprised the following : (i) 25 µg soluble PspA alone, (ii) 25 µg PspA administered with 50 µL Imject Alum, (iii) 5 µg PspA encapsulated into 250 µg 50:50 CPTEG:CPH nanoparticles + 20 µg soluble PspA, (iv) 5 µg PspA encapsulated into 250 µg 20:80 CPH:SA nanoparticles + 20 µg soluble PspA, (v) 1 µg soluble PspA alone, (vi) 1 µg PspA administered with 50 µL Imject Alum, (vii) 1 µg PspA encapsulated into 50 µg 50:50 CPTEG:CPH nanoparticles, (viii) 0.5 µg PspA encapsulated into 25 µg 50:50 CPTEG:CPH nanoparticles + 25 µg blank 50:50 CPTEG:CPH nanoparticles + 0.5 µg soluble PspA, or (ix) saline control. Blood was collected from mice *via* saphenous vein and serum was isolated following centrifugation (10,000 rcf for 10 min) at days 17 or 21 DPI. Serum was stored at −20°C until analysis could be performed.

**Table 1:** 25 µg Pneumococcal surface protein A (PspA)-containing single dose vaccine treatments groups.

Formulation	sPspA (μg)	ePspA (μg)
50:50 CPTEG:CPH PspA Nanovaccine	20	5
20:80 CPH:SA PspA Nanovaccine	20	5
sPspA + Alum	25	0
sPspA	25	0
Saline	0	0

*Separate groups of mice were immunized s.c. and all formulations were delivered in 100 µL total volume. Soluble PspA is represented as sPspA, and nanoparticle-encapsulated PspA is represented as ePspA*.

**Table 2:** One-microgram pneumococcal surface protein A (PspA)-containing single dose vaccine treatments groups.

Formulation	sPspA (μg)	ePspA (μg)
PspA Nanovaccine	0	1
PspA Nanovaccine + sPspA	0.5	0.5
sPspA + Alum	1	0
sPspA	1	0
Saline	0	0

*Separate groups of mice were immunized s.c. and all formulations were delivered in 100 µL total volume. Soluble PspA is represented as sPspA and nanoparticle-encapsulated PspA is represented as ePspA*.

### *S. pneumoniae* Challenge

*S. pneumoniae* strain A66.1, of PspA family 1, clade 2 was used for challenge and grown as previously described (56, 57). Briefly, the bacteria was grown at 37°C for 18 h in filter-sterilized Todd-Hewitt broth with 0.5% yeast extract, and stored at −80°C at a concentration of 1 × 10^8^ CFU/mL. Inoculum was diluted to a concentration of 2.5 × 10^4^ CFU/mL in sterile PBS and 100 µL was administered intraveneously at 24 DPI. Mice were monitored three times per day for the duration of the challenge (14 days). Body temperatures and body condition scores were also recorded once per day. Mice were sacrificed when determined to be moribund or at 14 days post-challenge.

### Anti-PspA Antibody Titer

Anti-PspA antibody titers were determined *via* an ELISA as described previously (37). Briefly, high-binding Costar 590 EIA/RIA microtiter plates (Corning) were coated overnight at 4°C with 0.5 µg/mL PspA. The blocking buffer for this assay comprised 2.5% (w/v) powdered milk dissolved in PBS-T and incubated for 2 h at 56°C to inactivate any endogenous phosphatase activity. PspA-coated plates were incubated with blocking solution for 2 h at room temperature before being washed three times with PBS-T. Serum obtained from immunized mice was added to the first well of a row at a dilution of 1:200 and serially diluted in PBS-T containing 1% (v/v) goat serum. Each serum sample was tested in duplicate. Following incubation overnight at 4°C, plates were washed three times with PBS-T after which secondary antibody was added at a dilution of 1 µg/mL. The secondary antibody used in these studies was alkaline phosphatase-conjugated goat anti-mouse IgG heavy and light chain (Jackson ImmunoResearch) and was incubated on the plates for 2 h at room temperature. Plates were then washed three times with PBS-T and alkaline phospatase substrate (Fisher Scientific, Pittsburgh, PA, USA) was added at a concentration of 1 mg/mL dissolved in 50 mM sodium carbonate, 2 mM magnesium chloride buffer at pH 9.3 for colorimetric development. Plates were analyzed after 30 min using a SpectraMax M3 microplate reader at a wavelength of 405 nm. Titer is reported as the reciprocoal of serum dilution at which optical density value was at least twice that of the saline group average plus two SDs.

### Shelf Stability of Stored PspA Nanovaccine

Shelf stability of the PspA nanovaccine was assessed *via* ELISA and lethal challenge. 50:50 CPTEG:CPH nanoparticles encapsulating PspA were taken out of the freezer (−80°C) and stored as a dry powder formulation at 25°C (room temperature) in a glass desiccator containing drierite for 60 days prior to immunization. Soluble PspA was stored at 4°C and combined with Imject Alum prior to immunization. A separate group of nanoparticles were kept in the freezer over the course of this experiment as a freezer-stored standard control. Following room temperature storage, vaccine efficacy was evaluated by immunizing mice with a nanovaccine dose equivalent to 1 µg PspA per animal from one of the storage conditions above, soluble PspA adsorbed to Alum, or the saline control and assessing antibody titer *via* ELISA 21 DPI (see “Anti-PspA Antibody Titer” for details). Following lethal challenge with 2,500 CFU of *S. pneumoniae* (see *S. pneumoniae* challenge for details), survival was assessed over the next 24 days.

### Statistical Analyses

Statistical analyses were performed using the Gehan–Breslow–Wilcoxon test and the one-way analysis of variance using Tukey’s multiple comparisons test. A *p*-value ≤0.05 was considered statistically significant. All analyses were performed using GraphPad Prism v. 7.0 software.

## Results

### Synthesis and Characterization of PspA-Encapsulated Polyanhydride Nanoparticles

**Figure 1:**
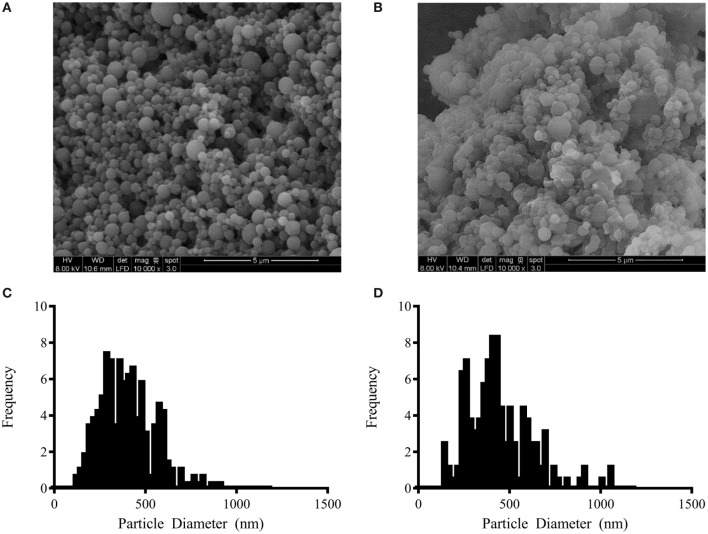
Pneumococcal surface protein A nanovaccine characterization. Representative scanning electron photomicrographs of **(A)** 2% PspA-loaded (w/w) 20:80 CPH:SA nanoparticles and **(B)** 2% PspA-loaded (w/w) 50:50 CPTEG:CPH nanoparticles (scale bar = 5 µm). **(C)** Particle size distribution of 2% PspA-loaded (w/w) 20:80 CPH:SA nanoparticles (422 ± 163 nm). **(D)** Particle size distribution of 2% PspA-loaded (w/w) 50:50 CPTEG:CPH nanoparticles (455 ± 175 nm).

The nanoparticle chemistries chosen for the current study were based on 50:50 CPTEG:CPH and 20:80 CPH:SA copolymers, consistent with our previous work, in which these chemistries were shown to provide protein structural stability and maintain the functional activity of PspA following release (37). The nanoparticles were synthesized using solid/oil/oil emulsion (54). The PspA release kinetics from these formulations are described in our previous work (37). Scanning electron photomicrographs of the nanoparticles revealed similar sizes for the two formulations, with sizes of 455 ± 175 and 422 ± 163 nm for 50:50 CPTEG:CPH and 20:80 CPH:SA nanoparticles, respectively (Figure 1). Zeta potential measurements of empty 20:80 CPH:SA and 50:50 CPTEG:CPH nanoparticles revealed that the particles had a negative surface charge, with zeta potentials of −41.9 ± 0.9 and −33.1 ± 5.1 mV, respectively, consistent with previous publications (45, 48, 58).

### Immunization with PspA-Based Nanovaccines Induces Protective Antibody Response

**Figure 2:**
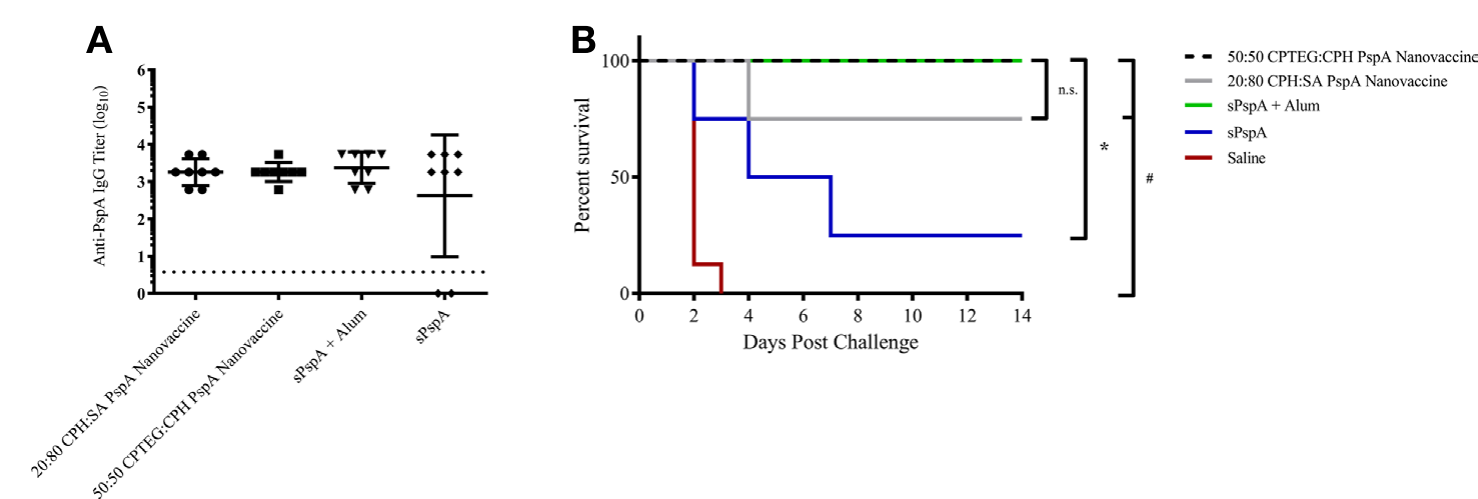
Pneumococcal surface protein A (PspA) nanovaccines are capable of initiating protective immune responses. **(A)** Serum was collected from immunized mice (see Table 1) at 17 days post-immunization (DPI) and analyzed for total anti-PspA IgG (data is represented as log_10_) using ELISA. Dashed line represents background anti-PspA IgG levels in serum from the saline-immunized mice. **(B)** *S. pneumoniae* challenge was performed at 24 DPI by i.v. administration of 2,500 CFUs of A66.1 strain and survival was monitored for 14 days post-challenge. * indicates significance (*p* ≤ 0.0025) compared to sPspA alone and ^#^ indicates significance (*p* ≤ 0.001) compared to saline-treated mice. Data are representative of one independent experiment containing *n* = 8 mice per treatment group.

Previously, it was demonstrated that mice vaccinated with PspA-based nanovaccines induced a robust humoral response; however, protective efficacy was not evaluated (37). In the current studies, separate groups of CBA/CaHN mice (*n* = 8 per group) were immunized subcutaneously as follows: (i) 25 µg soluble PspA alone, (ii) 25 µg PspA administered with Imject Alum, (iii) PspA-containing 50:50 CPTEG:CPH nanoparticles, (iv) PspA-containing 20:80 CPH:SA nanoparticles, or (v) saline (Table 1). Both the nanovaccine formulations contained 5 µg of encapsulated PspA along with 20 µg of soluble protein, as shown in Table 1. Anti-PspA total IgG titers were evaluated at 17 DPI and there were no significant differences in titer between vaccinated groups (Figure 2A). Mice were challenged with a lethal dose of *S. pneumoniae* strain A66.1 at 24 DPI and survival was assessed over a period of 2 weeks. All PspA naïve (saline) mice succumbed to infection within 4 days, while all the immunized animals showed prolonged survival following challenge. At 14 days post-challenge, only 25% of the (non-adjuvanted) soluble PspA-immunized animals survived, whereas adjuvanting the protein with alum provided 100% protection. Immunization with the 20:80 CPH:SA nanovaccine resulted in 87% survival, while 100% of the animals immunized with 50:50 CPTEG:CPH nanovaccine were protected, as shown in Figure 2B. The survival data, in correlation with the antibody titers indicate that not only is PspA an effective immunogen, but induces protective immunity when delivered with an adjuvant. Though both nanoparticle chemistries protected animals from lethal challenge compared to saline, the 50:50 CPTEG:CPH PspA nanovaccine performed significantly (*p* ≤ 0.008) better than soluble PspA (i.e., sPspA) alone. Based on these results, we selected the 50:50 CPTEG:CPH nanovaccine as our lead candidate for further evaluation.

### PspA Nanovaccine Demonstrates Dose-Sparing Capabilities

**Figure 3:**
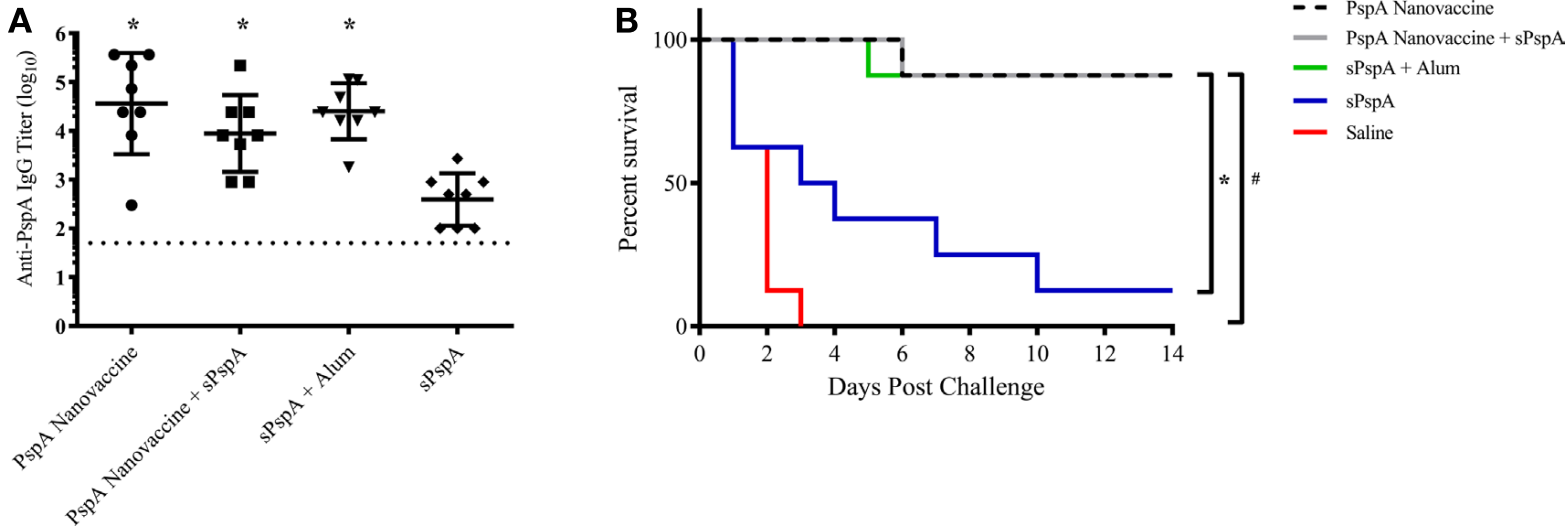
Nanovaccines with 25-fold reduction in total protein provided protection against lethal challenge. **(A)** Serum was collected from immunized mice (see Table 2) at 21 days post-immunization (DPI) and analyzed for total anti-PspA IgG (data is represented as log_10_) using ELISA. Dashed line represents background anti-PspA IgG levels in the serum from the saline-immunized mice. * indicates significance (*p* ≤ 0.008) compared to sPspA alone. **(B)** *S. pneumoniae* challenge was performed at 24 DPI by i.v. administration of 2,500 CFUs of A66.1 strain and survival was monitored for 14 days. * indicates significance (*p* ≤ 0.003) compared to sPspA alone and # indicates significance (*p* ≤ 0.0002) compared to saline-treated mice. Data are representative of one independent experiment containing *n* = 8 mice per treatment group.

Rational design of vaccines using subunit proteins involves considering total cost of the end product, as well as protein availability that potentially impacts vaccine shortages. Our previous work has shown that encapsulating a lowered dose of antigen into polyanhydride nanoparticles can induce a robust immune response (43). In the current studies, the ability of the nanovaccine to provide dose-sparing (i.e., 25-fold reduction) was evaluated by immunizing separate groups of CBA/N mice (*n* = 8 per group; see Table 2) subcutaneously using the following regimens: (i) 1 µg PspA encapsulated into 50:50 CPTEG:CPH nanoparticles (PspA Nanovaccine), (ii) 0.5 µg PspA encapsulated into 50:50 CPTEG:CPH nanoparticles plus 0.5 µg soluble PspA (PspA Nanovaccine + Soluble PspA), (iii) 1 µg soluble PspA administered with Imject Alum, (iv) 1 µg soluble PspA alone, and (v) saline alone (i.e., naïve control). Serum was collected at 21 DPI and analyzed for total anti-PspA IgG. Mice immunized with the nanovaccines or PspA + Alum showed significantly (*p* ≤ 0.008) higher titers to PspA compared to soluble PspA alone (Figure 3A). Survival rates following lethal challenge showed significantly (*p* ≤ 0.001) higher survival (87.5%) in mice immunized with both the nanovaccine formulations compared to animals immunized with soluble PspA (12%) (Figure 3B). Animals immunized with PspA adjuvanted with Alum performed similarly to the animals receiving the nanovaccine formulations. There were no observable differences in disease severity between the immunized animals, as measured by body condition scores and temperature (data not shown). In addition, soluble PspA administered in conjunction with 100 µg of empty 50:50 CPTEG:CPH nanoparticles, performed similarly to other adjuvanted groups, further indicating the ability for PspA to initiate a protective immune response when adjuvanted properly (data not shown). No significant differences in survival were observed between mice immunized with PspA fully encapsulated within the nanoparticles (i.e., PspA Nanovaccine) and animals that received half of the protein as a soluble bolus (i.e., PspA Nanovaccine + Soluble PspA). Eliminating the use of soluble PspA in the nanovaccine is beneficial when designing a shelf-stable dry powder formulation for the long-term storage of a PspA nanovaccine.

### PspA Nanovaccine Maintains Efficacy following Room Temperature Storage

**Figure 4:**
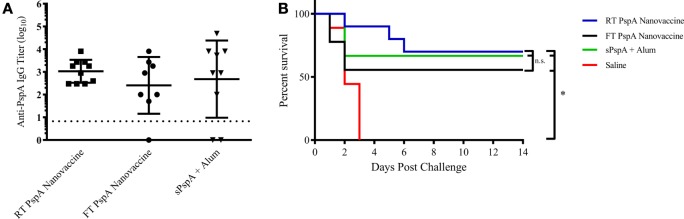
Room temperature-stored Pneumococcal surface protein A (PspA) nanovaccine maintains efficacy after 60 days. **(A)** Serum was collected from groups of immunized mice at 21 days post-immunization (DPI) with vaccine stored at different conditions as indicated and analyzed for total anti-PspA IgG (data are represented as log_10_) using ELISA. Dashed line represents background anti-PspA IgG levels in the serum from the saline-immunized mice. **(B)** *S. pneumoniae* challenge was performed at 24 DPI by i.v. administration of 2,500 CFUs of A66.1 strain and survival was monitored for 14 days. * indicates significance (*p* ≤ 0.001) compared to saline-treated mice. Data are representative of one independent experiment containing *n* = 8–10 mice per treatment group.

To evaluate the potential for a room temperature stored (i.e., non-refrigerated) PspA nanovaccine, 1 µg of PspA was encapsulated within 50 µg of 50:50 CPTEG:CPH nanoparticles (i.e., 2 wt.% loading) and stored at room temperature (25°C) and standard storage conditions (−80°C), as detailed in the Section “Materials and Methods” and as previously described (28). The PspA encapsulated nanovaccine was stored at −80°C (freezer stored) or at 25°C for 60 days. The soluble PspA was stored at 4°C and mixed with Alum prior to immunization. Following storage, separate groups of mice (*n* = 8–10 per group) were immunized subcutaneously with the PspA nanovaccine stored at the different conditions or with alum-adjuvanted PspA and serum was collected and analyzed at 21 DPI. Storage conditions did not have a significant effect on anti-PspA IgG titers, with all of the PspA nanovaccines performing similarly (Figure 4A). Mice were then challenged with a lethal dose of *S. pneumoniae* at 24 DPI and survival was monitored for 2 weeks. All of the non-immunized mice succumbed to challenge within 4 days post challenge, while the nanovaccine-immunized mice presented with 60–70% survival with a single dose (Figure 4B). Once again, no significant differences were observed between the survival rates of mice immunized with the PspA nanovaccine stored at various conditions and those immunized with soluble PspA adjuvanted with Alum. These data indicate that the PspA nanovaccine is efficacious when stored both in the freezer or at room temperature for time periods up to 60 days and performs similarly to refrigerated PspA adjuvanted with Alum.

## Discussion

*Streptococcus pneumoniae* is a causative agent of debilitating bacterial pneumonia. While many existing vaccines have proven to be valuable in reducing overall global disease burden, none are without limitations. Newly developed vaccine strategies against *S. pneumoniae* have identified several novel antigens, including PspA. Anti-PspA antibodies have been observed, following natural colonization or infection in children (59, 60). Other studies have shown success against lethal intranasal or septic *S. pneumoniae* challenge by vaccinating with PspA, which further indicates its potential as an efficacious vaccine immunogen (22, 61).

Aluminum hydroxide (i.e., Alum)-based adjuvants are widely used because of their ability to promote Th2 immune responses (62). However, Alum-based vaccines are associated with higher levels of immunization site tissue reactogenicity (63, 64). Immunization-site tenderness and pain are the most commonly reported symptoms following immunization with vaccines containing Alum. Prevalence of these adverse reactions has been found to be directly associated with increased protein dose administered and decreased age of the vaccinated individuals (65–67). Previous work with polyanhydride nanovaccines has shown low-level tissue site inflammation and toxicity making them a safe alternative to traditional vaccine adjuvants (32, 33, 41). Therefore, nanovaccines may represent a safe and efficacious alternative to Alum-based vaccines, which have limitations associated with administration site reactogenicity and only promote humoral immune responses against PspA.

Work from other laboratories has shown limited success using nanotechnology-based vaccine platforms with PspA in various animal models though, to date, none of these studies have shown protection against lethal challenge with single-dose immunization (68–70). Previous work from our laboratories has indicated that encapsulation of PspA into polyanhydride nanoparticles is capable of retaining protein structure and function, which enabled initiation of a humoral immune response (37). Building upon these studies, PspA was encapsulated within both 50:50 CPTEG:CPH and 20:80 CPH:SA nanoparticle formulations in the current work. Both formulations produced nanoparticles of similar size and size distribution (Figure 1). The size, morphology, and size distribution of these particles were similar to those previously reported for these formulations (28, 30, 37, 42). Following subcutaneous immunization of mice with a single dose of either formulation, anti-PspA IgG antibodies were produced, in agreement with our previous findings (37). Following lethal septic challenge, both nanovaccine formulations (containing 25 µg PspA) were capable of conferring protective immunity in mice, with 87.5% survival in 20:80 CPH:SA nanovaccine-immunized mice and 100% protection in those that received the 50:50 CPTEG:CPH nanovaccine (Figure 2). Based on the increased protection and higher antibody titers observed following immunization with a single dose of the 50:50 CPTEG:CPH nanovaccine formulation, this chemistry was selected as the lead candidate nanovaccine.

Reducing the amount of protein necessary per dose can greatly reduce the cost associated with using a recombinant protein vaccine, thereby allowing for the production and storage of more doses. Most of the published reports evaluating PspA-based vaccine success immunized mice with a minimum of 5 µg of antigen plus an adjuvant and required multiple boosts (56, 61, 71, 72). Indeed, a recent report demonstrated complete protection in mice receiving 0.5 µg of PspA adjuvanted with poly(I:C) intranasally; however, the immunization schedule required three doses to provide protection (71). The need for a single dose vaccination regimen cannot be overstated from a patient compliance standpoint because according to the WHO, an estimated 29% of children aged 1 month–5 years die each year from vaccine preventable diseases, citing non-compliance with the immunization regimen as a key reason for lack of successful national vaccination programs (73). Therefore, single-dose vaccines can aid in improving patient compliance, reduce cost, and improve national immunization rates worldwide. Recent work from our laboratories has demonstrated that 25 µg of ovalbumin encapsulated into polyanhydride microparticles was able to elicit robust antibody titers equivalent to a 16× larger soluble dose of ovalbumin while 25 µg of soluble ovalbumin alone was unable to elicit detectable antibody responses (43). In order to corroborate these results with PspA, the dose of antigen evaluated was reduced 25-fold to 1 µg. Immunization with the 1 µg PspA-based 50:50 CPTEG:CPH nanovaccine formulation, both with and without soluble PspA, induced production of high anti-PspA IgG titers (Figure 3A), with very little reduction in overall antibody titer compared to the previous study with a 25 µg dose of PspA (i.e., data in Figure 2). Antibody titers were similar between mice immunized with 1 µg of soluble PspA administered with Alum or with the nanovaccine formulations, with the highest overall titers observed in mice immunized with the fully encapsulated nanovaccine formulation. When compared to soluble protein adjuvanted with Alum, mice immunized with the 50:50 CPTEG:CPH nanovaccine were similarly protected from lethal challenge. Furthermore, 87.5% of the animals challenged with the reduced dose PspA nanovaccine (with or without soluble PspA) were protected (Figure 3B). These data demonstrate that reducing the protein dose by 25-fold had little effect on survival, with both the fully encapsulated PspA nanovaccine and the nanovaccine administered with a soluble bolus providing significantly higher survival following lethal challenge, compared to soluble protein alone.

By eliminating the soluble protein component from vaccine formulations, nanovaccines can be stored as a dry powder outside of refrigeration with the potential for long-term shelf storage, thereby circumventing the cold chain. As it currently stands, all prequalified WHO vaccines require refrigeration (73). Nanovaccines may enable next-generation vaccines to be stored without the need for refrigeration (i.e., eliminating the cold chain), allowing for vaccines to be sent to remote locations in need for vaccination, resulting in reduced medical costs and productivity losses associated with disease (74). Previous work from our laboratories demonstrated that nanovaccines encapsulating PspA were able to retain protein structure and activity following release, and a nanovaccine encapsulating the Bacillus anthracis protective antigen (PA) released biologically and immunologically functional protein following storage at both room temperature and above after 4 months (28, 37). By contrast, aluminum salt-based adjuvants have been reported to alter the structure of proteins and decrease thermal stability following adsorption (75), and previous work from our laboratories has shown that PA adsorbed to alum lost its functional activity when stored for 4 months at refrigerated conditions or above (28). Following storage at room temperature for 60 days, the PspA nanovaccine was shown to be efficacious, as measured by antibody titer and survival (Figure 4). PspA adsorbed to alum performed similarly, however, because PspA is a stable protein with coiled structure, it is likely that differences may be observed between the efficacy of the nanovaccine and alum-based formulations following storage for an extended period of time, or at elevated temperature, and should be evaluated in future studies (22). As such, this work indicates that the PspA nanovaccine formulation can be stored as a dry powder for at least 60 days while maintaining its immunogenicity.

In summary, this work describes the design of a safe next-generation PspA-based pneumonia nanovaccine with dose-sparing, protective immunity, and enhanced room temperature storage. These studies are promising first steps with respect to establishing the polyanhydride nanovaccine platform as an alternative to traditional vaccines in terms of reduced cost (enabled by the dose sparing and single dose capabilities) and difficulties associated with transportation and storage of vaccines in developing countries (enabled by the superior room temperature storage capabilities).

## Ethics Statement

This study was carried out in accordance with the recommendations of the Iowa State University Institutional Animal Care and Use Committee. The protocol was approved by the Iowa State University Institutional Animal Care and Use Committee.

## Author Contributions

DW-M, SH, and SK performed the experiments and analyzed the data. MW and BN, together with DW-M, SH, and SK, designed the experimental plan. All the authors participated in writing the manuscript.

## Conflict of Interest Statement

The authors declare that the research was conducted in the absence of any commercial or financial relationships that could be construed as a potential conflict of interest.
